# Chagas Cardiomiopathy: The Potential of Diastolic Dysfunction and Brain Natriuretic Peptide in the Early Identification of Cardiac Damage

**DOI:** 10.1371/journal.pntd.0000826

**Published:** 2010-09-21

**Authors:** Ana Garcia-Alvarez, Marta Sitges, María-Jesús Pinazo, Ander Regueiro-Cueva, Elizabeth Posada, Silvia Poyatos, José Tomás Ortiz-Pérez, Magda Heras, Manel Azqueta, Joaquim Gascon, Ginés Sanz

**Affiliations:** 1 Thorax Clinic Institute, Hospital Clínic, Institut d'Investigacions Biomèdiques August Pi i Sunyer (IDIBAPS), University of Barcelona, Barcelona, Spain; 2 Fundación Centro Nacional de Investigaciones Cardiovasculares Instituto de Salud Carlos III, Madrid, Spain; 3 Institut d'Investigacions Biomèdiques August Pi i Sunyer (IDIBAPS), Barcelona, Spain; 4 Barcelona Centre for International Health Research (CRESIB), Hospital Clinic, Institut d'Investigacions Biomèdiques August Pi i Sunyer (IDIBAPS), University of Barcelona, Barcelona, Spain; National Institutes of Health, United States of America

## Abstract

**Introduction:**

Chagas disease remains a major cause of mortality in several countries of Latin America and has become a potential public health problem in non-endemic countries as a result of migration flows. Cardiac involvement represents the main cause of mortality, but its diagnosis is still based on nonspecific criteria with poor sensitivity. Early identification of patients with cardiac involvement is desirable, since early treatment may improve prognosis. This study aimed to assess the role of diastolic dysfunction, abnormal myocardial strain and elevated brain natriuretic peptide (BNP) in the early identification of cardiac involvement in Chagas disease.

**Methodology/Principal Findings:**

Fifty-four patients divided into 3 groups—group 1 (undetermined form: positive serology without ECG or 2D-echocardiographic abnormalities; N = 32), group 2 (typical ECG abnormalities of Chagas disease but normal 2D-echocardiography; N = 14), and group 3 (regional wall motion abnormalities, left ventricular [LV] end-diastolic diameter >55 mm or LV ejection fraction <50% on echocardiography; N = 8)—and 44 control subjects were studied. Patients with significant non-cardiac diseases, other heart diseases and previous treatment with benznidazol were excluded. The median age was 37 (20–58) years; 40% were men. BNP levels, longitudinal and radial myocardial strain and LV diastolic dysfunction increased progressively from group 1 to 3 (p for trend <0.01). Abnormal BNP levels (>37 pg/ml) were noted in 0%, 13%, 29% and 63% in controls and groups 1 to 3, respectively. Half of patients in the undetermined form had impaired relaxation patterns, whereas half of patients with ECG abnormalities suggestive of Chagas cardiomyopathy had normal diastolic function. In group 1, BNP levels were statistically higher in patients with diastolic dysfunction as compared to those with normal diastolic function (27±26 *vs.* 11±8 pg/ml, p = 0.03).

**Conclusion/Significance:**

In conclusion, the combination of diastolic function and BNP measurement adds important information that could help to better stratify patients with Chagas disease.

## Author Summary

Chagas disease remains a major cause of morbidity and mortality in several countries of Latin America and has become a potential public health problem in countries where the disease is not endemic as a result of migration flows. Cardiac involvement represents the main cause of mortality, but its diagnosis is still based on nonspecific criteria with poor sensitivity. Early identification of patients with cardiac damage is desirable, since early treatment may improve prognosis. Diastolic dysfunction and elevated brain natriuretic peptide levels are present in different cardiomyopathies and in advanced phases of Chagas disease. However, there are scarce data about the role of these parameters in earlier forms of the disease. We conducted a study to assess the diastolic function, regional systolic abnormalities and brain natriuretic peptide levels in the different forms of Chagas disease. The main finding of our investigation is that diastolic dysfunction occurs before any cardiac dilatation or motion abnormality. In addition, BNP levels identify patients with diastolic dysfunction and Chagas disease with high specificity. The results reported in this study could help to early diagnose myocardial involvement and better stratify patients with Chagas disease.

## Introduction

Chagas disease, a major cause of morbidity and mortality in several countries of Latin America [1], has become a potential public health problem in countries where the disease is not endemic as a result of migration flows [2], [3], [4]. Chagas cardiomyopathy is the most serious form of the chronic phase of the disease and represents the major cause of mortality in these patients. For this reason, accurate diagnosis of cardiac involvement is critical. However, Chagas disease remains a neglected disease [5] and the diagnosis of Chagas cardiomyopathy is still based on simple and nonspecific criteria including an increased cardiothoracic ratio (>0.5) or ECG abnormalities such as complete right bundle-branch block, left anterior hemiblock, complete left bundle-branch block, as well as other conduction and rhythm disturbances [6], [7]. Echocardiography refined the diagnosis of Chagas cardiomyopathy, and regional wall motion abnormalities, reduced left ventricular ejection fraction (LVEF) <50% and increased left ventricular (LV) end-diastolic diameter >55 mm are now included as diagnostic criteria in some publications [8], [9]. In spite of that, the sensitivity of these parameters is far from perfect and they may indeed misclassify patients with early myocardial involvement into the undetermined form, as conventional 2D echocardiography only detects advanced myocardial involvement. On the other hand, patients without cardiac disease but having one of the described as typical but unspecific ECG findings could be considered to have Chagas cardiomyopathy. Therefore, a more accurate classification model, particularly to identify patients with early cardiac involvement from the undetermined form would be desirable, since an early treatment and closer follow-up might be beneficial on these patients [8].

Analysis of diastolic function by echocardiography, cardiac magnetic resonance (CMR) and several biomarkers, including brain natriuretic peptide (BNP) and inflammation markers, have emerged as useful tools in the diagnosis and monitoring of heart failure in different conditions. In fact, BNP has recently been included in the guidelines for the diagnosis and management of congestive heart failure [10]. Comprehensive evaluation of diastolic dysfunction and myocardial strain imaging has provided more accuracy and sensitivity to detect early myocardial involvement in different cardiomyopathies [11], [12]. However, these methods have been seldom utilized in characterizing patients with established Chagas cardiomyopathy [13], [14], [15], and its role in the identification of cardiac involvement in the earlier phases of the disease is unclear [16], [17], [18].

We conducted a prospective study aimed to analyze the added value of different techniques in identifying cardiac involvement in the undetermined stage of Chagas disease. Diastolic function, natriuretic peptide and inflammatory markers levels in different phases of the disease were measured and correlated with longitudinal and radial myocardial strain and delayed enhancement on CMR to find out their value in improving the stratification of patients with Chagas disease.

## Methods

### Study Population

A cross-sectional analysis was performed in a prospective cohort of consecutive adult patients evaluated at our Institution from January 2008 to June 2009. Diagnosis of Chagas disease was based on a clinical record compatible with the epidemiology of the disease (individuals from endemic zones of Chagas disease) and microbiologic diagnosis by any combination of at least two positive commercial serological tests using different antigens: ELISA using a *T. cruzi* lysate (Ortho-Clinical Diagnostics®), ELISA with recombinant antigens (BioELISA Chagas®, Biokit S.A.) and indirect immunofluorescence (Inmunofluor Chagas, Biocientifica®). In an attempt to avoid factors that could have an effect on diastolic function, myocardial strain, natriuretic peptides or inflammatory markers levels, patients with severe non-cardiac diseases, prior diagnosis of heart disease from other etiology (ischemic, hypertensive or alcoholic), hypertension, diabetes mellitus, active infections by other causal agent or previous treatment with benznidazol were excluded. All patients gave written consent for inclusion. The research protocol was approved by the Ethics Committee of our institution.

Patients meeting the inclusion criteria were categorized into 4 groups: Group 0 (control group, subjects from endemic areas with negative serology for Chagas disease); Group 1 (patients in the undetermined form of Chagas disease defined as those with positive serology of Chagas disease without any abnormal ECG finding, normal LV dimensions and LV global and regional systolic function with conventional 2D echocardiography); Group 2 (patients with typical ECG abnormalities of cardiac involvement by Chagas disease such as complete right bundle-branch block and/or left anterior hemiblock, complete left bundle-branch block, ventricular premature beats, primary abnormalities of ventricular repolarization, electrically inactive zones, low voltage QRS, sinus bradycardia <50 beat/min, advanced atrioventricular block or cardiac pacemaker, but normal LV dimensions and global and regional systolic function by 2D-echocardiography); and Group 3 (patients with Chagas cardiomyopathy with any regional wall motion abnormality and/or LV end-diastolic diameter >55 mm and/or LVEF <50% by 2D-echocardiography).

Clinical examination, blood analysis including ions, creatinin, inflammatory markers and natriuretic peptides levels as well as a comprehensive 2D-echocardiogram with diastolic function and myocardial strain analysis were obtained from all patients. CMR studies were performed in an unselected sample of patients with Chagas disease (Groups 1–3).

### Biochemical measurements

Measurements of plasmatic levels of endothelin 1, tumor necrosis factor-α (TNFα), interleukin 6 (IL-6), atrial natriuretic peptide (ANP) and BNP levels was carried out through peripheral venous puncture, after a 30 minutes rest, and quantified using commercially available kits. BNP levels were measured using a fully automated two-site sandwich BNP immunoassay on an Advia Centaur (Siemens Diagnostics, Zurich, Switzerland). Minimum sensitivity and upper limit of normal values are 2 and 37 pg/ml respectively for the BNP assay. The precision of this technique is 1.8–4.3%.

### Imaging techniques

Echocardiographic studies were performed with a commercially available system (VIVID 7, General Electrics; Milwaukee, WI). Images were digitally stored for later off-line analysis with a commercial software package (EchoPac, General Electrics; Milwaukee, WI). LV volumes and LVEF were calculated using the modified Simpson rule (biplane method). Left atrium area was measured at the end-ventricular systole excluding the confluences of the pulmonary veins and the left atrium appendage. LV volumes and left atrium area were indexed to body surface area. Analysis of diastolic function was performed evaluating the mitral inflow pattern with pulsed Doppler: E and A waves and deceleration time of the E wave (DT); the pulmonary vein flow and the mitral annulus velocities with Doppler Tissue Imaging (Em and Am). Patients were classified according to diastolic function patterns (normal, impaired relaxation or stage I, pseudonormal or stage II and restrictive pattern or stage III) following current recommendations [19]. The ratio of early diastolic mitral flow velocity to early diastolic mitral annulus velocity (E/Em) was used as a surrogate of LV filling pressures. LV segmental myocardial longitudinal 2D-strains were acquired from two and four-chamber apical views and radial strains from the parasternal short axis view at the level of the papillary muscles. Averages of longitudinal and radial strains were obtained by dividing the sum of all segmental strains by the number of analyzed segments. Images were optimized to obtain a frame rate >50 fps.

CMR studies were performed using a 1.5 T clinical scanner (Signa CV HDxt, General Electric, Milwaukee WI). Functional assessment was studied with a standard cine steady-state free precession sequence and delayed-enhanced images were acquired using a gradient-echo segmented inversion recovery technique, 10 minutes after intravenous administration of gadodiamide at a dose of 0.2 mmol/Kg (Omniscan, GE Healthcare, Madrid). LV end-diastolic, end-systolic volumes and LVEF were calculated using Mass 4.0.1 software analysis (MEDIS, The Netherlands). All echocardiographic and CMR analyses were performed by experienced independent observers blinded to BNP measurements and clinical data.

### Statistical analysis

Continuous baseline variables were expressed as mean±SD or median (interquartile range) values depending on normality assessed by the Shapiro-Wilks test. Categorical variables were expressed as total number (percentages) and compared between groups using Chi-square test or Fisher's test. Differences in continuous variables were analyzed using either ANOVA test or Kruskall Wallis test depending on variable distribution. Post-hoc analysis using either T-test or Wilcoxon test corrected by Bonferroni method was carried out to detect differences between each pair of groups. Trends in continuous variable changes across Chagas's disease severity and diastolic function impairment were analyzed using either ANOVA test for trend or Jonckheere-Terpstra test. Correlations between natriuretic peptides and LV volumes and LVEF were assessed using Pearson coefficient. Receiver-operating characteristic (ROC) curves were constructed to estimate the accuracy of BNP and ANP to detect any grade of diastolic dysfunction. Statistical analysis was performed with SPSS 15.0®.

## Results

A total of 98 consecutive subjects were included. Median age was 37 (range 20 to 58) years and 40% were men. There were 44 subjects in Group 0, 32 patients in Group 1, 14 in group 2, and 8 patients in group 3. Clinical characteristics, hemodynamic data, and levels of inflammatory markers and natriuretic peptides are shown in Table 1. Creatinin and sodium plasmatic levels were normal for all patients. Only two patients were under pharmacological treatment: one patient in group 1 was treated with betablockers and one patient in group 3 was under angiotensin converting enzyme inhibitors. There were no differences in clinical and hemodynamic characteristics between groups except for the New York Heart Association (NYHA) functional class. The majority of patients were in NYHA functional class I; no patient had NYHA functional class III or IV. TNFα levels were higher in group 1 as compared to group 0 and a significant trend towards increasing levels was observed according worsening clinical forms of Chagas disease. Abnormally high BNP levels (>37 pg/ml) were noted in 0%, 13%, 29% and 63% of patients in groups 0, 1, 2 and 3, respectively. BNP and ANP levels in group 3 were significantly elevated as compared to those in groups 0 and 1. There were no statistically significant differences in IL6 and endothelin 1 levels between groups. IL6 levels were undetectable in 68% of patients.

**Table 1:** Demographic and hemodynamic characteristics and blood markers of control individuals and patients in the undetermined and cardiac forms.

*Variable*	*Group 0 Control (N = 44)*	*Group 1 Undetermined (N = 32)*	*Group 2 ECG findings (N = 14)*	*Group 3 Abnormal echo (N = 8)*	*P*	*P for trend*
Age (years)	34.0 (11.5)	36.8 (14.6)	42.7 (17.4)	40.9 (8.6)	0.02	<0.01
Gender (male)	18 (41%)	13 (34%)	6 (43%)	4 (50%)	0.80	NA
Smoking habit	6 (14%)	2 (6%)	1 (7%)	2 (25%)	0.42	NA
Hypercholesterolemia	2 (5%)	2 (6%)	2 (14%)	1 (13%)	0.60	NA
Systolic BP (mmHg)	106±11	113±14	113±15	113±14	0.09	0.05
Diastolic BP (mmHg)	66±8	69±10	70±11	68±16	0.31	0.15
Heart rate (bpm)	60.0 (9.0)	65.0 (10.0)	60.0 (9.0)	56.0 (23.0)	0.31	0.94
NYHA FC II	0 (0%)	0 (0%)	2 (14%)	3 (38%)	0.01	NA
Endothelin (pmol/L)	6.8 (2.4)	6.3 (3.2)	6.0 (2.4)	6.7 (5.0)	0.35	0.66
IL6 (pg/ml)	1.7±3.2	11.3±28.3	0.0±0.0	22.2±54.0	0.09	0.12
TNFα (pg/ml)	3.0 (6.5)	7.0 (5.8)*	7.0 (4.0)	9.5 (7.3)	<0.01	<0.01
BNP (pg/ml)	10.3 (10.2)	12.3 (17.1)	15.3 (31.0)	43.6 (190.0)* †	<0.01	<0.01
BNP>37 pg/ml	0 (0%)	4 (13%)	4 (29%)	5 (63%)	<0.01	NA
ANP (pg/ml)	23.8 (11.0)	26.5 (15.0)	28.0 (11.3)*	54.0 (39.8)* †	<0.01	<0.01

Continuous variables are expressed as mean ± standard deviation or median (interquartile range); categorical variables are expressed as number of patients (%).

BP: blood pressure; NYHA FC =  New York Heart Association functional class; TNFα: tumour necrosis factor alpha; BNP: brain natriuretic peptide; ANP: atrial natriuretic peptide. NA: non applicable (categorical variables); P =  p value between groups; P for trend: p value for a trend in continuous variable changes across Chagas's disease severity.

*Statistically significant differences versus group 0.

**†:** Statistically significant differences versus group 1.

Echocardiographic data regarding LV volumes, LVEF, LV myocardial strains and diastolic function are shown in Table 2. Patients in the undetermined form (group 1) showed no differences in LV dimensions or global LVEF as compared to the control group but had significantly reduced Em and lengthened DT (0.14±0.03 m/s vs. 0.16±0.03 m/s and 238.5 ms vs. 200 ms, respectively; p<0.001 for both).

**Table 2:** Echocardiographic parameters of control individuals and patients in the undetermined and cardiac forms.

*Variable*	*Group 0 Control (N = 44)*	*Group 1 Undetermined (N = 32)*	*Group 2 ECG findings (N = 14)*	*Group 3 Abnormal echo (N = 8)*	*P*	*P for trend*
LVEDV (ml/m^2^)	59.5 (13.2)	56.2 (14.3)	68.1 (13.8)	93.7 (25.4)* † ‡	<0.01	0.02
LVESV (ml/m^2^)	23.0 (7.4)	21.7 (6.6)	28.4 (8.7)	52.0 (15.1)* † ‡	<0.01	0.02
LA (cm/m^2^)	7.8 (2.1)	8.6 (2.8)	9.1 (1.9)	10.8 (5.2)*	<0.01	<0.01
LVEF (%)	64.5 (5.0)	65.0 (4.8)	60.0 (9.0)	41.0 (12.5)* † ‡	<0.01	<0.01
E (m/s)	0.85±0.13	0.78±0.17	0.76±0.11	0.72±0.17	0.05	<0.01
A (m/s)	0.51 (0.2)	0.60 (0.2)	0.59 (0.3)	0.50 (0.26)	0.30	0.51
Em (m/s)	0.16±0.03	0.14±0.03*	0.12±0.04*	0.09±0.04* †	<0.01	<0.01
Am (m/s)	0.09 (0.04)	0.09 (0.03)	0.09 (0.04)	0.08 (0.04)	0.60	0.58
E/Em	5.0 (1.9)	5.9 (2.0)	7.1 (3.4)	7.9 (3.9)* †	<0.01	<0.01
DT (ms)	200.0 (45.0)	238.5 (62.0)*	251.5 (140.0)*	284.5 (172.7)*	<0.01	<0.01
Average LS (%)	19.3 (3.8)	19.3 (2.9)	17.1 (5.0)	15.8 (6.9)* † ‡	<0.01	<0.01
Average RS (%)	49.3 (22.5)	39.8 (36.2)	40.2 (22.8)	16.0 (13.2)* † ‡	<0.01	<0.01

Continuous variables are expressed as mean ± standard deviation or median (interquartile range); categorical variables are expressed as number of patients (%).

LVEDV: left ventricular end-diastolic volume indexed to body surface area; LVESV: left ventricular end-systolic volume indexed to body surface area; LA: left atrium area indexed to body surface area; LVEF: left ventricular ejection fraction; E: early diastolic mitral flow velocity; A: late diastolic mitral flow velocity; Em: early mitral annulus diastolic tissue velocity; Am: late mitral annulus diastolic tissue velocity; E/Em: ratio of early diastolic mitral flow velocity to early diastolic mitral annulus velocity; DT: deceleration time of the E wave; LS: myocardial longitudinal strain; RS: myocardial radial strain. P =  p value between groups; P for trend: p value for a trend in continuous variable changes across Chagas's disease severity.

*Statistically significant differences versus group 0.

**†:** Statistically significant differences versus group 1.

**‡:** Statistically significant differences versus group 2.

When patients were classified according to the diastolic function pattern, 50% of patients in groups 1 and 2 had an impaired relaxation pattern. On contrast, every patient in group 3 had a certain degree of diastolic dysfunction (Table 3). Two (5%) subjects in group 0 had impaired relaxation pattern. Natriuretic peptides and LV myocardial strain averages were progressively different as diastolic dysfunction severity increased (p for trend <0.01) (Table 4). The accuracy of BNP and ANP to detect any grade of diastolic dysfunction, as assessed by the area under the ROC curve, was 0.73, 95%CI 0,60–0.85 for BNP and 0.70; 95%CI 0.58 – 0.81 for ANP. In addition, both BNP and ANP significantly correlated with LV end-diastolic volumes (r = 0.35; p = 0.001 and r = 0.26; p = 0.013, respectively), LV end-systolic volumes (r = 0.44; p<0.001 and r = 0.36; p<0.001 respectively) and LVEF (r = −0.44; p<0.001 and r = −0.44; p<0.001, respectively).

**Table 3:** Diastolic function in patients with undetermined and cardiac forms of Chagas disease.

	Group 1 Undetermined (N = 32)	Group 2 ECG findings (N = 14)	Group 3 Abnormal echo (N = 8)
Normal (N = 23)	16 (50%)	7 (50%)	0 (0%)
Impaired relaxation (N = 25)	16 (50%)	5 (36%)	4 (50%)
Pseudonormal (N = 6)	0 (0%)	2 (14%)	4 (50%)

Data are expressed as number of patients (%).

**Table 4:** Serum and myocardial strain according to diastolic function classification in patients with Chagas disease.

	Normal (N = 23)	Impaired relaxation (N = 25)	Pseudonormal (N = 6)	P	P for trend
BNP (pg/ml)	10.1 (9.2)	18.7 (39.3) †	43.4 (245.0) *	<0.01	<0.01
ANP (pg/ml)	26.0 (12.0)	30.0 (25.0)	55.0 (69.8) *	<0.01	<0.01
Edothelin1 (pmol/L)	5.7 (4.2)	6.7 (2.3)	7.3 (6.3)	0.15	0.05
IL6 (pg/ml)	2±6.1	14.0±32.7	29.0±64.8	0.16	0.06
TNFα (pg/ml)	7.0 (4.2)	6.5 (6.0)	10.0 (9.5)	0.71	0.47
Average LS (%)	18.4 (3.0)	18.7 (4.6)	14.4 (6.5) *	0.04	<0.01
Average RS (%)	40.2 (37.8)	31.2 (34.1)	14.8 (16.5) *	0.01	<0.01

Data are expressed as mean ± standard deviation or median (interquartile range).

BNP: brain natriuretic peptide; ANP: atrial natriuretic peptide; TNFα: tumour necrosis factor alpha. P =  p value between groups; P for trend: p value for a trend in continuous variable changes across diastolic dysfunction impairment.

*Statistically significant differences between pseudonormal pattern and Normal pattern.

**†:** Statistically significant differences between impaired relaxation pattern and Normal pattern.

**Figure 1:**
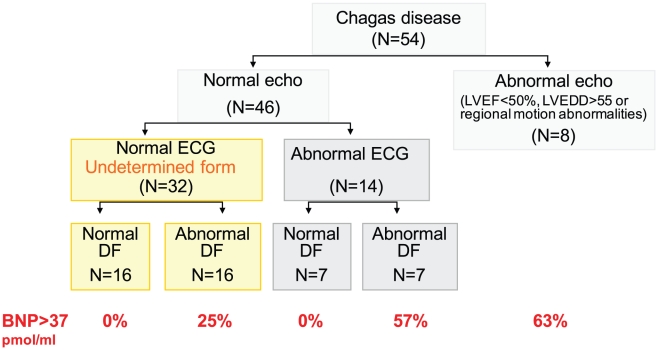
Distribution of patients according to the conventional 2D-echocardiography, ECG and diastolic function and prevalence of pathologic levels of BNP (% of patients). Echo =  conventional 2D-echocardiography; ECG =  electrocardiogram; DF =  diastolic function (assessed by echocardiography); BNP =  Brain natriuretic peptide. Normal echo means normality in LV dimensions and LV global and regional systolic function assessed with conventional 2D echocardiography.

When only patients in the undetermined form of the disease (group 1) were considered, BNP levels were higher in patients with diastolic dysfunction compared to those with normal diastolic pattern (27±26 versus 11±8 pg/ml, respectively, p = 0.03). A similar result was obtained in group 2, thus, a trend towards increasing BNP levels was observed from normal diastolic pattern to abnormal relaxation and pseudonormal pattern (11±4, 37±36 and 41±3, pg/ml respectively, p = 0.06). The areas under the ROC curves for BNP and ANP to detect mild diastolic dysfunction in patients in the undetermined form were 0.69; 95%CI 0,49–0.89 for BNP and 0.62; 95%CI 0.43–0.82 for ANP. Additionally, every patient with abnormally high levels of BNP (>37 pg/ml) had diastolic dysfunction (Figure 1).

**Figure 2:**
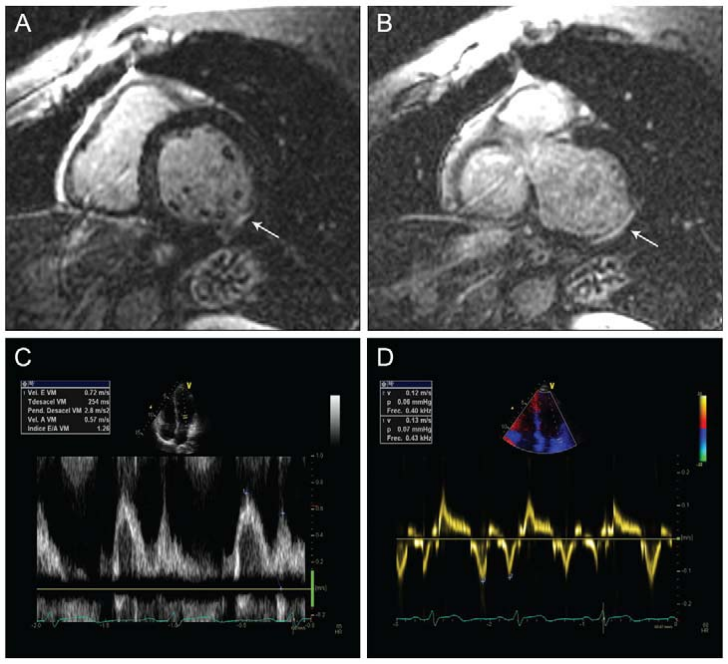
Example of a patient in the undetermined form of Chagas disease with delayed enhancement on cardiac magnetic resonance and abnormal diastolic function. Short axis delayed enhanced CMR images (panels A & B) showing focal linear hyperenhancement in the basal inferolateral segment (red arrows). Panels C and D depict Doppler mitral inflow and myocardial tissue velocity imaging pattern consistent with impaired diastolic dysfunction in the same patient.

**Figure 3:**
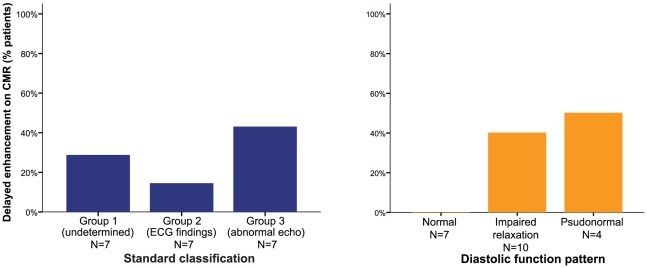
Delayed enhancement on CMR (observed cases/N) in Chagas diseases patients. A Conventional classification. B Classification based on diastolic function. CMR =  Cardiac Magnetic Resonance; ECG =  electrocardiogram; Echo =  conventional 2D-echocardiography.

A CMR was performed in 21 Chagas disease patients, 7 patients in each group (groups 1–3) according to the standard classification [20], [21]. Two (28%) patients in group 1 (Figure 2), 1 (14%) patient in group 2 and 3 (43%) patients in group 3 had gadolinium delayed enhancement compatible with scar or fibrosis. However, when these patients were classified according to the diastolic function pattern, none with normal diastolic function had delayed enhancement, whereas 40% of patients with impaired relaxation pattern and 50% with pseudonormal pattern showed delayed enhancement (Figure 3).

## Discussion

The main finding of our investigation is that diastolic dysfunction occurs before any LV dilatation, regional or global systolic abnormalities or significant increased filling pressures as assessed by E/Em ratio. In this regard, we found significant reduction in Em and lengthening in DT in patients in the undetermined form compared to control individuals, in spite of preserved LV volumes and systolic function. The fact that all the rest of the echocardiographic measurements remained similar in both the undetermined form and control individuals, suggests that Em and DT could be the most sensitive echocardiographic parameters to detect cardiac involvement by Chagas disease. In addition, BNP levels identify patients with diastolic dysfunction among those in the undetermined form of Chagas disease with high specificity. The study also confirmed the fact that as the disease progresses from the undetermined form to Chagas cardiomyopathy with abnormal 2D-echocardiography, including enlargement of LV volumes and a deterioration of global LV function, diastolic function deteriorates. This is supported also by the fact that global LV strain, both longitudinal and radial, also progressively decreases along with diastolic function impairment, suggesting the existence of myocardial damage, despite preserved LVEF.

Previous studies analyzing diastolic function in the undetermined form of Chagas disease have shown conflicting results (15–21). Barros et al. were the first to report early LV diastolic dysfunction in patients in the undetermined form of Chagas disease as they demonstrated a lengthening of DT and isovolumic relaxation time [16]. Cianciulli et al. similarly observed that transmitral Doppler flow allowed to identify early abnormalities of diastolic function in patients with normal ECG and conventional 2D-echocardiogram [17]. On the other hand, Pazin-Filho et al. also focused on patients in the undetermined form and showed that patients with normal global and segmental LV systolic function by 2D echocardiography did not show any abnormality of diastolic function [18]. However, in a meticulous evaluation of this small study a trend for higher LA volumes, DT lengthening and Em reduction could be observed among groups, and therefore, an insufficient statistical power might have contributed to the negative result.

Despite being only performed in a small subgroup of patients, the results of CMR also sustain that diastolic dysfunction may be the first manifestation of myocardial involvement, before systolic dysfunction occurs as it happens in other cardiomyopathies. Therefore, the comprehensive analysis of diastolic dysfunction could be more sensitive in terms of early diagnosis of myocardial involvement compared to the standard classification as it was shown that some patients with normal ECG and normal conventional 2D-echocardiography had myocardial fibrosis detected by delayed enhancement, whereas no patient with normal diastolic function had enhancement on CMR. A prior study showed that delayed enhancement in CMR can be present in up to 20% of patients who are in the undetermined form of the disease [22], suggesting that this technique has an extended value for the diagnosis in early stages of cardiac involvement. However, CRM availability is limited particularly in areas where Chagas disease is endemic. The association between a normal diastolic function by echo and absence of fibrosis on CMR has to be confirmed in larger series.

Nevertheless, although diastolic function analysis seems to have a high sensitivity, it has to be acknowledged that the complexity of diastolic dysfunction measurements may preclude its use in large populations, especially in underdeveloped geographical areas. In this regard, the initial screening with BNP determination could be helpful, especially with the use of simplified, point-of-care kits. In fact, every patient with abnormally high BNP levels had diastolic dysfunction in our study (Figure 1).

Natriuretic peptides have been shown to be involved in the pathogenesis of Chagas disease in animal experiments [23], [24] and some clinical studies have reported that BNP levels are increased in Chagas cardiomyopathy [25], [26], [27] and correlates with prognosis [28]. Ribeiro et al. demonstrated a high specificity with moderate sensitivity for BNP to detect LVEF≤40% in infected patients with an abnormal ECG or chest X-ray [25]. In a second study, the same group reported that BNP levels correlated with LV dimensions and LVEF in patients with Chagas disease and also that patients with mild degree of cardiac dysfunction, defined as no more that minor alterations in their echocardiography, had intermediate BNP levels compared to control individuals and patients in the cardiac form of the disease [27]. In a third study, they compared the diagnostic accuracy of the combination of BNP plasmatic levels and ECG vs. the standard strategy (ECG and chest X-ray) to detect LVEF ≤40% and demonstrated a significant improvement in specificity although the new strategy had less sensitivity [26]. However, even though the correlation between BNP levels and systolic dysfunction in Chagas disease has been well described, few studies have correlated BNP levels and diastolic function in Chagas disease [14], [29]; indeed, all of them have been done in patients in the cardiac form of Chagas disease. Barbosa et al [14] evaluated 59 patients with dilated cardiomyopathy due to Chagas disease and reported a marked elevated concentration of the amino-terminal portion proBNP specifically in patients with a restrictive diastolic pattern. Oliveira et al [29] evaluated 36 patients, all of them with diffuse or segmental ventricular motion abnormalities, and described a significant correlation between BNP and E/E' ratio in the inferior wall. To our knowledge there are no studies that have specifically assessed the association between BNP and diastolic function in patients in the undetermined form. In our population, the accuracy of BNP to detect any degree of diastolic dysfunction was good (area under curve of 0.73); additionally, the ability to detect mild diastolic dysfunction in the group of patients in the undetermined form was also good (area under the curve of 0.69). The specificity of BNP levels >37 pg/ml to detect mild diastolic dysfunction in patients in the undetermined form of Chagas disease was 100%. The power of ANP levels to detect diastolic dysfunction was slightly inferior”.

Different from ANP, BNP is mainly secreted in the ventricles in response to wall stress, ischemia or fibrosis. Indeed, myocardial fibrosis has been described to strongly trigger BNP synthesis [30]. Chagas cardiomypathy is a predominately fibrogenetic cardiomyopathy. Cardiac fibrosis is evident even in early stages of the disease [22], [31]. This fact might explain why BNP levels could be elevated even in patients with normal NYHA functional class, normal LVEF and ventricular filling pressures, and supports the idea that BNP levels measurement could be useful to early detect cardiac involvement in Chagas disease. Enhanced fibrosis, compared with other cardiomyopathies, could also explained higher BNP levels in patients with Chagas disease as compared to patients with cardiac disease of different etiologies in the same NYHA functional class [32].

Our study also aimed to assess the plasmatic levels of TNFα, IL6 and endothelin 1, in patients with different clinical forms of Chagas cardiomyopathy. These biomarkers have been described to be elevated in Chagas cardiomyopathy [33], [34], [35]. We found statistically significant elevated levels of TNFα in patients in the undetermined form as compared to control individuals and a significant trend towards increasing levels was observed along clinical severity groups. Our finding is in concordance with previously published literature suggesting heart inflammation in patients with Chagas disease even in the absence of heart failure. Talvani et al [35] had previously reported higher TNFα levels in patients with severe Chagas cardimyopathy; in this study although TNFα levels were slightly higher in patients in the undetermined form as compared to those in healthy individuals, no statistically significant differences were reached. Similarly, in our study, a trend towards greater IL6 levels could be also observed along with progressive clinical severity and diastolic function impairment. However, as this measurement was undetectable in a significant proportion of patients, its interpretation is limited. The use of high-sensitivity kits for IL6 detection could be valuable in this context. Finally, our study failed to demonstrate differences in plasmatic levels of endothelin 1 in the different forms of Chagas disease. A privious study reported elevated endothelin plasmatic levels in patients with Chagas cardiomyopathy [33]. However, in this study seropositive patients had similar endothelin plasmatic levels than control individuals, and the group of patients in the undetermined form had even lower levels than controls. Therefore, the usefulness of plasmatic measurement of endothelin is not clear and more studies are required to clarify its role in the clinical evaluation of patients with Chagas disease.

The main limitation of our study is its cross-sectional design and, consequently progression of cardiomyopathy could not be evaluated. Longitudinal studies in Chagas disease are difficult due to the slow progression of the disease and the frequent change of residency that particularly patients who live in non-endemic areas have, making follow-up difficult.

In conclusion, the initial screening with measurement of BNP, an easy test with high specificity for LV damage, combined with a comprehensive analysis of diastolic function that contributes with high sensitivity seems to be a more accurate strategy to early diagnose LV involvement in Chagas disease. Our findings could help to better stratify patients with Chagas disease. The association between normal diastolic function, normal BNP levels, absent fibrosis on CMR and no progression of the disease warrants confirmation in larger and prospective longitudinal studies. Also, new studies are required to demonstrate that early treatment and closer follow-up slow the disease progression and consequently improve the prognosis in these patients.

## Supporting Information

Checklist S1 STROBE checklist.
(0.08 MB DOC)
