# Supplementary material for: Chagas Cardiomiopathy: The Potential of Diastolic Dysfunction and Brain Natriuretic Peptide in the Early Identification of Cardiac Damage
Source: PLoS Negl Trop Dis. 2010 Sep 21;4(9):e826. doi: 10.1371/journal.pntd.0000826 (PMC2943653; doi:10.1371/journal.pntd.0000826)
Supplement: Checklist S1 — STROBE checklist. (0.08 MB DOC) [file pntd.0000826.s001.doc]

STROBE Statement—checklist of items that should be included in reports of observational studies

|  | | Item No | Recommendation |
| --- | --- | --- | --- |
| **Title and abstract** | | 1 | (*a*) Page 1 |
|  |  |  | (*b*) Page 2 |
| Introduction | | | |
| Background/rationale | | 2 | Page 4 |
| Objectives | | 3 | Page 5 1^st^ paragraph |
| Methods | | | |
| Study design | | 4 | Page 5-6 |
| Setting | | 5 | Page 5 2^nd^ paragraph |
| Participants | | 6 | (*a*) Page 4 |
|  |  |  |  |
| Variables | | 7 | Page 4-5 |
| Data sources/ measurement | | 8* | Page 4-5 |
| Bias | | 9 | Page 13 (limitations) |
| Study size | | 10 | n/a |
| Quantitative variables | | 11 | Page 7 |
| Statistical methods | | 12 | Page 7 |
| Results | | | |
| Participants | 13* | Page 8 | |
| Descriptive data | 14* | Page 8 Table 1 | |
| Outcome data | 15* | Page 8-9 | |
| Main results | 16 | Page 8-9 Table 1-4 | |
| Other analyses | 17 | n/a | |
| Discussion | | | |
| Key results | 18 | Page 9 1^st^ paragraph discussion | |
| Limitations | 19 | Page 13 | |
| Interpretation | 20 | Page 11,13 | |
| Generalisability | 21 | Page 13 | |
| Other information | | | |
| Funding | 22 | Submission form | |

*Give information separately for cases and controls in case-control studies and, if applicable, for exposed and unexposed groups in cohort and cross-sectional studies.

**Note:** An Explanation and Elaboration article discusses each checklist item and gives methodological background and published examples of transparent reporting. The STROBE checklist is best used in conjunction with this article (freely available on the Web sites of PLoS Medicine at http://www.plosmedicine.org/, Annals of Internal Medicine at http://www.annals.org/, and Epidemiology at http://www.epidem.com/). Information on the STROBE Initiative is available at www.strobe-statement.org.
